# An RNA Switch of a Large Exon of Ninein Is Regulated by the Neural Stem Cell Specific-RNA Binding Protein, Qki5

**DOI:** 10.3390/ijms20051010

**Published:** 2019-02-26

**Authors:** Yoshika Hayakawa-Yano, Masato Yano

**Affiliations:** Division of Neurobiology and Anatomy, Graduate School of Medical and Dental Sciences, Niigata University, 757, Ichibancho, Asahimachidori, Chuo-ku, Niigata, Niigata 951-8510, Japan; yoshika@med.niigata-u.ac.jp

**Keywords:** Ninein, Quaking, RNA-binding protein, HITS-CLIP, neural development

## Abstract

A set of tissue-specific splicing factors are thought to govern alternative splicing events during neural progenitor cell (NPC)-to-neuron transition by regulating neuron-specific exons. Here, we propose one such factor, RNA-binding protein Quaking 5 (Qki5), which is specifically expressed in the early embryonic neural stem cells. We performed mRNA-SEQ (Sequence) analysis using mRNAs obtained by developing cerebral cortices in *Qk* (*Quaking*) conditional knockout (cKO) mice. As expected, we found a large number of alternative splicing changes between control and conditional knockouts relative to changes in transcript levels. DAVID (The Database for Annotation, Visualization and Integrated Discovery) and Metascape analyses suggested that the affected spliced genes are involved in axon development and microtubule-based processes. Among these, the mRNA coding for the Ninein protein is listed as one of Qki protein-dependent alternative splicing targets. Interestingly, this exon encodes a very long polypeptide (2121 nt), and has been previously defined as a dynamic RNA switch during the NPC-to-neuron transition. Additionally, we validated that the regulation of this large exon is consistent with the Qki5-dependent alternative exon inclusion mode suggested by our previous Qki5 HITS-CLIP (high throughput sequencing-cross linking immunoprecipitation) analysis. Taken together, these data suggest that Qki5 is an important factor for alternative splicing in the NPC-to-neuron transition.

## 1. Introduction

Alternative pre-mRNA splicing is a major mechanism for producing diverse proteins from a single gene, and drives cellular differentiation [1,2,3,4]. In the brain, pre-mRNA splicing generates transcriptome complexity between neural subtypes and developmental stages [5,6], and its mis-regulation is often associated with neurological diseases [7,8]. Several reports show that alternative splicing dynamics regulate neural development through RNA-binding proteins (RNABPs), such as Ptbp1/2, Srrm4, RbFoxs, RBM4, Qki5, and Nova2, in conjunction with their direct RNA switches [9,10,11,12,13,14,15,16]. For example, *Dab1* splicing is involved in neocortical radial migration [14,17], *Ank3* splicing switch is involved in the assembly of the axon initial segment [18], *Elavl4* splicing switch changes splicing activity [19], and *Nin* and *Flna* splicing are involved in the maintenance of apical neural progenitor cells (NPCs) and neuronal differentiation during NPC-to-neuron transition [16]. Of particular interest are the different roles that Ninein plays in neurons and NPCs, e.g., regulation of axonal development in neurons [20] and formation of centrosome structure in neural stem cells [21]. Alternative splicing variants give rise to these functional differences of the Ninein protein between NPCs and neurons. These differing functions are well-characterized by differential subcellular protein localization. In NPCs, Ninein is localized to the centrosome by binding to the centrosomal proteins CEP170 and CEP250. In these cells, the Ninein protein includes domains encoded by a very large, internal alternative exon, exon 18, as well as exon 29bc. In neuronal cells, Ninein lacks exon 18 and includes exon 29a, and does not bind to the centrosomal proteins, as evidenced by its diffuse cytoplasmic localization [16]. In neuronal cells, exon 29a is included by the RNABPs RbFox1/2/3, but the regulation of exon 18 remains unclear. 

*Quaking* (*Qk*) gene generates three major alternative spliced mRNAs encodes three RNA-binding protein isoforms, Qki5, Qki6, and Qki7, which differ only in C-terminal regions [22]. In our previous study, we proposed Qki5 as a novel neural stem cell regulator in the developing neocortex, as evidenced by its highly specific expression in neural stem cells relative to Qki6 and 7 [11]. Qki5 governs alternative splicing events through three modes of pre-mRNA splicing regulation, as predicted by transcriptome analysis and HITS-CLIP, a method that map the protein-RNA interaction at the transcriptome-wide [23,24,25] and suppresses neuronal transcript levels in primary neural stem cell culture [11]. Although this evidence suggests that Qki5 is associated with NPC-to-neuron transition via pre-mRNA splicing, the splicing dynamics and specific targets of Qki5 in the developing neocortex remain unclear. In the present study, we performed mRNA-SEQ analysis using mRNAs obtained from the cortex of *Qk* conditional knockout (cKO) mice to examine Qki5-dependent RNA splicing dynamics as they relate to the NPC-to-neuron transition. We found several Qki5-dependent alternative splicing changes, including a very large exon18 of the *Nin* gene, which is strongly associated with the NPC-to-neuron transition. 

## 2. Results

### 2.1. The mRNAseq Analysis of Qk Conditional Knock-Out Cortices

**Figure 1:**
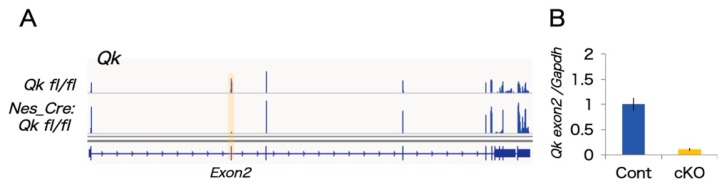
The mRNA-SEQ analysis from E14.5 cortices. (**A**) IGV (Integrative Genome Viewer) image showing mRNA expression of the *Qk* gene from control versus *Qk* cKO cortices. As expected, mRNA from cKO does not include exon 2 because of the loxP sites flanking exon 2 of the *Qk* gene for gene targeting to obtain conditional knockouts. (**B**) Bar graphs show the results of a qRT-PCR validation using control and *Qk* cKO cortices for *Qk* mRNA.

**Figure 2:**
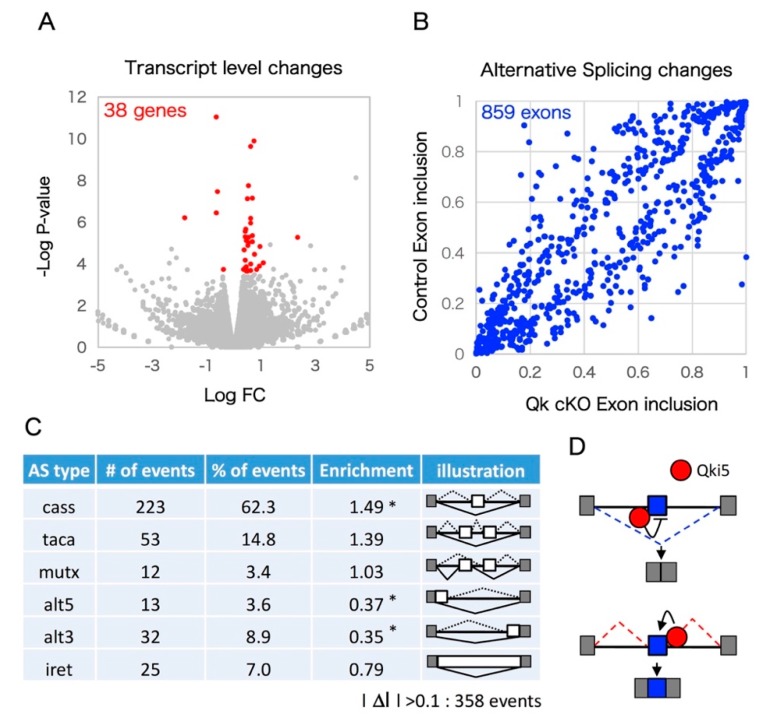
Pre-mRNA splicing defects in *Qk* conditional knockout cortex pre-mRNA splicing defects in Qk conditional knockout cortices. (**A**) Steady state of mRNA level between control and *Qk* cKO. Volcano plot diagrams shown. The significance (*p*-value) versus fold change is plotted on the *Y*-axis and *X*-axis, respectively. (**B**) Scatter plot of the exon inclusion of control versus *Qk* cKO mRNA-SEQ significant hits. Each point represents the mean obtained from two biological replicates for an individual alternative splicing event. (**C**) Qki5 regulates many types of alternative splicing patterns. Asterisk indicates significant enrichment by Fisher’s exact test (* *p* < 0.001). (**D**) Schematic illustration of the bidirectional Qki5 action on cassette exon type alternative splicing regulation. Note: cass: Cassette exon; taca: tandem cassette exon; mutx: mutually exclusive exon; alt5: alternative 5′ site; alt3: alternative 3′ site; iret: intron retention.

We performed unbiased mRNA-SEQ analysis to detect Qki-dependent transcriptomics in the developing neocortex. Embryonic day 14.5 mouse cortex RNA from two independent *Qkfl*/*fl* (control) and *Nestin*-cre:*Qkfl*/*fl* (*Qk cKO*) littermates was used to prepare mRNA libraries through polyA selection and 150 base pair (bp) paired-end sequencing using the Illumina (San Diego, CA, USA) HiSeq system. Sequence reads were aligned to the mouse genome (mm10) and an exon junction database using OLego, and gene expression levels and alternative splicing events were quantified using Quantas [26]. In total, about 80 million paired-end reads were obtained for the control and *Qk cKO* mRNA-SEQ samples. Over 10 million clusters included spliced junction reads among uniquely mappable regions (Table 1). Importantly, Exon 2 of *Qk* mRNA was greatly reduced in *Qk* cKO, indicating high efficiency of Cre recombination and low contamination of non-neural tissue (Figure 1A). This reduction was also confirmed by a quantitative RT-PCR assay (Figure 1B). We investigated mRNA-SEQ data to determine whether loss of the *Qk* gene affects steady-state levels of mRNA. In *Qk* cKO, steady-state levels of 15 transcripts were significantly upregulated, and 43 transcripts were significantly downregulated (EdgeR statistics; *p* < 0.01, FDR < 0.1) (Figure 2A and Supplemental Table S1). Following the removal of weakly expressed transcripts (RPKM < 1.0 in either control or *Qk* cKO), we found only 38 transcripts with significant changes (Figure 2A). This suggests the possibility that the nuclear isoform Qki5, which functions in pre-mRNA splicing rather than in regulating expression level, is expressed in embryonic cortices at higher levels than the other isoforms, Qki6 and Qki7, which play roles in steady-state level of mRNA abundance [11,27]. To further determine whether the *Qk cKO* affects pre-mRNA splicing events in the embryonic neocortex, we next analyzed mRNA-SEQ data at the exon junction level. We discovered 859 mRNA splicing defects, which could be due to changes in Qki-dependent RNA splicing events with significant differences relative to the control (Fisher’s exact test in Quantas: *p* < 0.01, FDR < 0.1) (Figure 2B). Among the 358 alternative splicing changes that included at least 0.1 of Δ*I* ratio between control and cKO, all alternative exon types (cassette type, tandem cassette type, mutually exclusive, 5′ASS, 3′ASS, and retained intron) were included. Notably, the cassette-type exon was significantly enriched compared with other types (enrichment score: 1.49) (Figure 2C). In addition, the changes of their cassette type exons are largely consistent with a position dependent alternative splicing mechanism, which we previously defined as evidenced by adding the information of Qki5-HITS-CLIP (high throughput sequencing-cross linking immunoprecipitation) result (Figure 2D, Supplemental Table S2 and [11]).

### 2.2. Enrichment Analyses Revealed that Qki Dependent Pre-mRNA Splicing Is Involved in Cytoskeleton-Related Pathways

**Figure 3:**
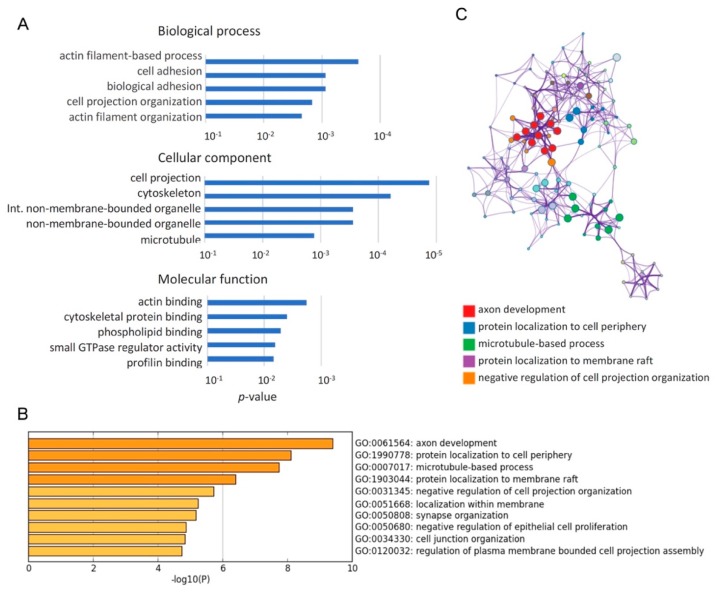
Cytoskeleton related genes were enriched among genes with Qki regulating alternative exons. (**A**) Gene ontology analysis using DAVID (The Database for Annotation, Visualization and Integrated Discovery) bioinformatics revealed that cytoskeleton-related terms were overrepresented in genes with Qki-regulated alternative exons. GO (Gene Ontology) terms ranked among the top 5 terms with *p*-value are represented in the bar graph. (**B**) Gene ontology analysis using Metascape revealed that cytoskeleton-related terms were over-represented in genes with Qki-regulated alternative exons. GO terms ranked among the top 10 terms with *p*-value are represented in the bar graph. (**C**) In Metascape analysis, significant terms were hierarchically clustered into a network tree based on Kappa-statistical similarities among their gene memberships. Each term is represented by a circle node, where its size is proportional to the number of input genes fall into that term, and its color represent its cluster identity. Terms with a kappa similarity score > 0.3 are linked by an edge.

To investigate how *Qk* deficiency affects biological processes in cortical development, we performed a gene ontology (GO) analysis for a list of 251 genes with differentially spliced exons between control and *Qk* cKO (l Δ*I* l > 0.1 and RPKM > 1) using DAVID (The Database for Annotation, Visualization and Integrated Discovery) bioinformatics resources (Figure 3A). This comprehensive analysis revealed that cytoskeleton-related GO terms, such as actin filament, cell adhesion, cell projection, and microtubule, are over-represented in the GO terms ranked among the top 5 terms. To further identify the biologically relevant pathways, we performed Metascape analysis using the same list as above. GO terms, such as axon development, protein localization to cell periphery, and microtubule-based process, were identified as statistically enriched ontology clusters among Qki-regulated alternative splicing events (Figure 3B,C and Table 2). Notably, *Flna* and *Nin*, both of which have biologically relevant exons related to the NPC-to-neuron transition [16], are repetitively listed in genes of cytoskeleton-related GO terms. These results suggest that Qki5 is an important factor in pre-mRNA splicing during NPC-to-neuron transition in the embryonic neural stem cells.

### 2.3. A Large Exon 18 of Nin Is Regulated by Neural Stem Cell Regulator Qki5

**Figure 4:**
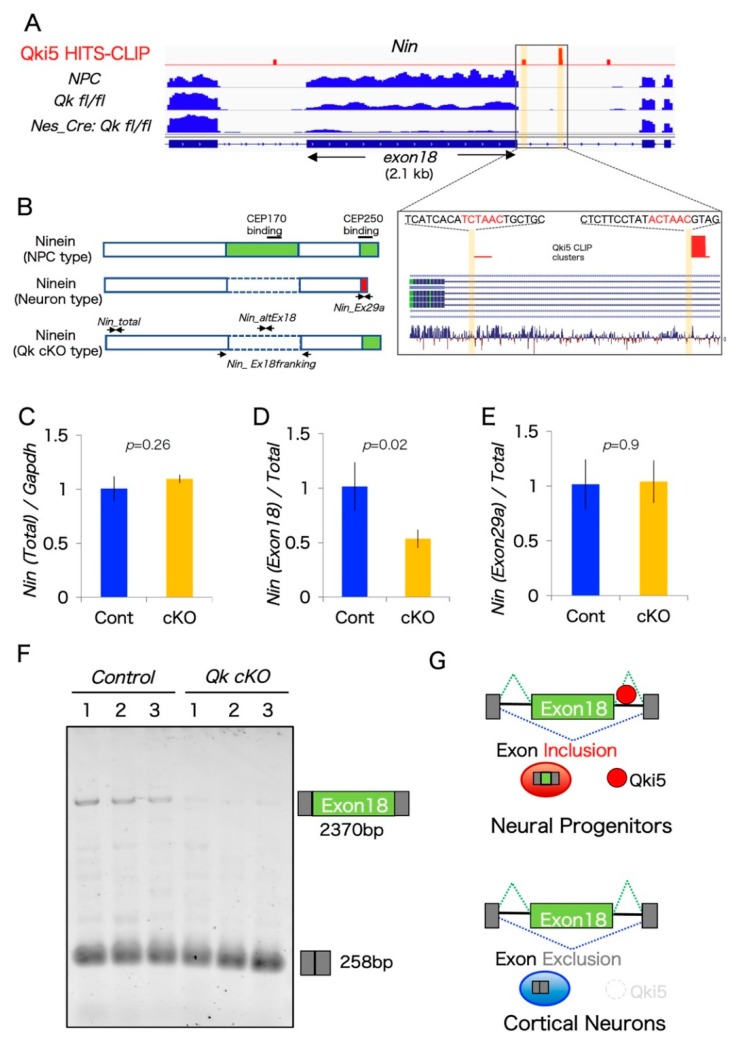
Ninein coding a large alternative exon is regulated by the Qki5 protein along with neuronal differentiation. (**A**) IGV image showing mRNA expression and Qki5 HITS-CLIP clusters along the *Nin* gene including the alternative large exon 18 and its flanking region from control (*Qk* *fl*/*fl*), *Qk* cKO cortices (*Nestin-cre:Qkfl/fl*), and cultured embryonic neural stem cells (NPC). Magnified view of *Nin* gene showing the Qki5-enhanced alternative exon and Qki5 CLIP clusters, including defined Qki5-binding site ACUAAC (highlighted in yellow). (**B**) Protein structure of NPC-Ninein, neuron-Ninein proteins, and NPC-neuron hybrid Ninein with centrosomal protein-binding sites. (**C**–**E**) The qRT-PCR analysis in control and *Qk* cKO cortices was performed to monitor the splicing of each alternative exon of *Nin*. (**F**) RT-PCR gel image of each pre-mRNA splicing of *Nin* exon 18 in control and *Qk* cKO, performed to monitor the splicing of alternative exons. (**Bottom panels**) Primers corresponding to each flanking exon were used to determine the expression of aberrantly processed mRNAs in *Qk* cKO. (**G**) Qki5-dependent pre-mRNA splicing model during NPC-to-neuron transition.

One previous report demonstrated that RNA switching exons from two genes, *Nin* and *Flna*, are associated with the NPC-to-neuron transition in neocortical development [16]. Interestingly, our aberrant pre-mRNA splicing list from *Qk* cKO includes alternative exons from both *Nin* and *Flna*. However, the Qki-dependent alternative splicing exon of *Flna* was different from the critical exon N of *Flna*, which contains a premature stop codon, thereby controlling Flna protein level through the nonsense-mediated decay pathway. By contrast, we found that a large alternative exon 18 of *Nin* was dramatically downregulated in *Qk* cKO cortices. Large exon 18 (>2.1 kb) is almost exclusively included in mouse and human NPCs, as well as in our previous mRNA-SEQ result from mouse neural stem cells, and completely excluded in cortical neurons (Figure 4A and [16]). Additionally, another exon 29bc of *Nin* is also specifically excluded in cortical neurons but included in NPCs [16]. Interestingly, these two exons, 18 and 29bc from *Nin* mRNA, encode the functional protein domains for association with the centrosomal proteins CEP170 and CEP250, respectively (Figure 4B). 

We next performed quantitative PCR validation analysis for the two *Nin* alternative splicing exons using three different biological replicates. The result from two PCR primer sets flanking the constitutive exons and exon 29a-specific did not show any differences between control and *Qk* cKO. By contrast, large exon 18 expression levels were significantly reduced in *Qk* cKO, reflecting mRNA-SEQ data (Figure 4A,C–E). To further confirm that large alternative exon 18 is expressed and lost in *Qk* cKO, we carried out semi-quantitative RT-PCR analysis. Importantly, this large exon (over 2.1 kb) is dramatically decreased in *Qk* cKO cortices (Figure 4F). In addition, we mapped Qki5 HITS-CLIP data, which we have previously performed using E14.5 mouse whole brain, around large exon 18. The Qki5 binding map indicated a significant peak at the intron downstream from alternative large exon 18 of *Nin*, which contained the clear Qki5-binding motif ACUAAC (Figure 4A). Given the mechanism of Qki5-dependent alternative pre-mRNA splicing, downstream binding of Qki5 to normal alternative cassette exons enhances exon inclusion [11]. In an additional gain-of-function study, we observed upregulation of large exon 18 of *NIN* in Qki5-over-expressed HEK293 cells, not Qki6 (Supplemental Figure S1). We conclude that Qki5 directly regulates a large exon 18 of *Nin*. Furthermore, our previous observation shows that Qki5 is almost exclusively expressed in NPC cells, when neurogenesis actively occurs in the embryo, but neurons do not express it [11]. This evidence supports the idea that Qki5 enhances NPC-Ninein expression via alternative splicing, and non-Qki5-expressing neurons express neuron-Ninein, suggesting a role of Qki5 in the NPC-to-neuron transition (Figure 4G).

## 3. Discussion

Using genetics and mRNA-SEQ analysis, we found that the RNA-binding protein Qki5 regulates a very large internal alternative exon 18 of *Nin*, which is related to the NPC-to-neuron transition. In addition, we proposed a mechanism by which the binding of Qki5 to a downstream intron promotes this large alternative exon. This mechanism was suggested by adding HITS-CLIP information, in which we previously demonstrated three modes of Qki5-dependent alternative splicing in neural stem cells.

Alternative splicing plays important roles in multiple aspects of cortical development. Indeed, defects of neurogenesis have been seen in several murine mutants of genes encoding splicing regulators, such as srrm4, ptbp1/2, Nova2, and Qki5 [11,28]. These RNABPs govern a huge number of target pre-mRNAs, including alternative exons and micro-exons, thereby contributing to multiple aspects of cortical development and complicated transcriptomics. However, a large number of alternative pre-mRNA mis-splicings in RNABP-null mice result in a pleiotropic phenotype [29,30,31]; therefore, it has been difficult to identify how the individual functions of these alternative isoforms contribute to cortical development. For example, a mutant deficient in the neuron-specific splicing factor, Nova, exhibited multiple deficits in the central nervous system, such as radial neuronal migration and axon guidance/synapse formation of neuro-muscular junctions. In vivo rescue experiments demonstrated that the alternative RNA switches *Dab1*, which is a major component of Reelin signaling, and *Agrin*, which is an inducer of clustering of AchRs, were involved in each biological event, respectively [14,32]. Recently, another example was reported in the context of embryonic neocortical development in mouse and human brain. Alternative splicing dynamics from two genes, *Nin* and *Flna*, control critical events, such as neural progenitor expansion and neuronal differentiation in neocortical development. Heterozygous null mutation of *FLNA* in females causes periventricular nodular heterotopia (PVNH) [33]. Interestingly, deep intronic *FLNA* mutation derepresses ectopic exon N inclusion, which has a premature stop codon and induces the nonsense-mediated decay pathway in NPCs, leading to decreased protein levels and mild brain malformation. This mutation- driven alternative exon N inclusion was associated with disruption of NPC factor PTBP1-dependent alternative splicing skipping. By contrast, the repertoire of protein products from the *Nin* gene is dynamically controlled by alternative RNA switching during the NPC-to-neuron transition. Ninein protein isoforms are thought to have different roles in the NPC-to-neuron transition due to their subcellular localization, with NPC-Nin exhibiting strong centrosomal localization mediated by the centrosomal protein CEP family, and neuronal-Nin diffusely localized to the cytoplasm because neuronal-Nin does not contain the C-terminal domain, which encodes a centrosomal localization signal [16]. Importantly, this exon switching is partially associated with neuronal splicing regulators RbFox1/2/3; however, the regulation of a very large exon 18 has been completely unresolved. The present study proposes Qki5 as a critical splicing regulator for the enhancement in the exon 18 of *Nin*.

We recently proposed a novel neural stem cell type splicing regulator, Qki5. Mutants of Qki5 show defects in cell-to-cell adhesion and mislocalization of the centrosomal protein γ-tubulin in neural stem cells, which could be mediating the beta-catenin pathway [11]. We performed unbiased mRNA-SEQ analysis using E14.5 *Nestin-cre*: *Qk* conditional knockout cortices. This analysis revealed a huge number of alternative splicing defects related to microtubule organization, including the *Nin* and *flna* transcripts. Interestingly, we found that Qki5 knockout resulted in exclusion of a very large exon 18 of *Nin*, thereby generating the NPC-neuron hybrid Ninein protein in neural stem cells. This hybrid Ninein protein might be involved in the phenotypic features of Qki5 conditional knockout mice because Qki5-null neural stem cells exhibit mislocalization of centrosomal protein γ-tubulin [11].

In this study, we focused on a larger alternative exon, *Nin* exon 18, which is over 2.1 kb. Previous bioinformatics data suggested that large internal exons over 1000 nt are a minor population, comprising about 0.5% of all internal exons, and only 4.7% of all human protein-coding genes include them [34]. The number of exons with a size greater than 2000 nt is dramatically decreased. A previous biochemical study that examined exons ranging from 50 to 1500 nt demonstrated that large exon size does not limit splicing [35]. Therefore, we proposed that larger alternative exon 18 is also regulated by standard cellular splicing machinery in combination with an additional cell-specific splicing factor, Qki5. Indeed, the present data demonstrate not only the reduction of *Nin* exon 18 inclusion in *Qk* conditional knockout cortices, but the mechanism by which the direct Qki5 action on downstream ACUAAC site enhances exon inclusion in a manner consistent with our previously defined position-dependent map of Qki5 functional exon regulation (Figure 4 and [11]). Moreover, exon 18 of *Nin* dramatically decreased at the NPC-to-neuron transition, which correlated with Qki5 expression patterns, as evidenced by our previous finding that Qki5 is specifically expressed in neural stem cells and lost in neuronal cells. These data collectively indicate that Qki5 can be added to the list of factors required for widespread alternative exon usage during NPC-to-neuron transition.

## 4. Materials and Methods 

### 4.1. Mice 

All of the animal experiments were conducted in compliance with the protocol reviewed by the Institutional Animal Care and Use Committee of Niigata University and approved by the President of Niigata University (approval code SA00191 approved on 5 April 2018). To generate embryonic conditional *Qk* knock-out mice, *Qk*^*flox*/*flox*^ mice were crossed with Nestin-Cre transgenic mice, as previously described [11]. To detect the Nestin transgenes and Cre recombinase, the following primers were used: Nes-F: 5′-AATCCTTCTTCGGGC TTCGG-3′ and Cre-R: 5′-TTGCGAACCTCATCACTCGT-3′. 

### 4.2. The mRNA Sequence and Data Analysis

Total RNA was obtained from three independent replicates of E14.5 mouse cortices from control and *Qk* conditional knockouts. The RNA was extracted using QIAzol (Qiagen; Venlo, The Netherlands) followed by the generation of mRNA libraries using Illumina TruSeq protocols for poly-A selection, fragmentation, and adaptor ligation according to the manufacturer’s instructions (TruSeq RNA Sample Prep Kit v2). The multiplexed libraries were sequenced as 150 nt pair end runs on an Illumina HiSeq 4000 system (Novogen, Pledran, France). Sequence reads were mapped to the reference mouse genome (mm10) using OLego splicing aligner (Zhang Laboratory, networking in the RNA world, https://zhanglab.c2b2.columbia.edu/index.php/OLego). Calculation of exon inclusion rate and differential exon inclusion rate between two groups (Δ*I*) were quantified using a Fisher exact test in the Quantas tool [26]. We used FDR < 0.1 and *p* < 0.01 as significant alternative changes. Differential expression statistics of mRNA abundance were performed with EdgeR [36].

### 4.3. Accession Numbers

The mRNA-SEQ data have been deposited in GEO under accession number GSE123927 (http://www.ncbi.nlm.nih.gov/geo/query/acc.cgi?acc=GSE123927) and also see GSE103248 for neural stem cells.

### 4.4. Enrichment Analyses

A dataset of 251 genes having alternative splicing changes between control and *Qk* cKO (lΔ*I* l > 0.1 and RPKM > 1) were subjected to enrichment analyses using DAVID bioinformatics resources 6.7 (https://david.ncifcrf.gov/) [37] and Metascape (http://metascape.org/gp/index.html) [38]. For both analyses, 12,490 genes (RPKM > 1) expressed in the mouse cortex were used as background genes. Functional enrichment was performed in three categories of GO terms, biological process (BP), molecular function (MF), and cellular component (CC), in DAVID. 

### 4.5. RT-PCR and qRT-PCR

RT-PCR analyses were performed as described elsewhere [39]. At least three biological replicates were used for quantitative PCR, which was performed with a StepOnePlus real-time PCR detection system (Applied Biosystems; Waltham, MA, USA). Mouse *Gapdh* gene were used as internal control, and results were calculated using the ΔΔCT method. Semi-quantitative long PCR assays were performed in a total volume of 25 µL containing optimized PCR buffer for KOD-plus-Neo (Toyobo; Tokyo, Japan). Visualized gel image with SyberGold (Invitrogen; Carlsbad, CA, USA) was obtained by iBright CL1000 system (Invitrogen).

### 4.6. Primers for qRT-PCR and RT-PCR 

Primers used for PCR experiments were as follows:

*Nin_Total*: (For Figure 4C)

F:5′-TGCAACAGACGCTACTCCAG-3′ R:5′-GTGATCTTCGTCCATAACGCTT-3′

*Nin_altExon18*: (For Figure 4D) 

F:5′-CCGAGAAAACTCCTGCCTTC-3′ R:5′-GTCTCCATCACCTCCTCCATTT-3′

*Nin_Exon29a*: (For Figure 4E)

F:5′-TGGGACATGTTGGAATCAAGTG-3′ R:5′-GCAAGCAGAAGTTTGTGACTGAA-3′

Nin_Ex18_flanking: (For Figure 4F)

F:5′-GGAGAAGGTGAGAGGCTTGG-3′ R:5′-CACAATTCTTCCTGAGAGCCATTT-3′

## Figures and Tables

**Table 1:** Statistics for mRNA-SEQ (Sequence).

Sample	Total Reads	Pair.gapless.bed	Mappable (%)	Junction Reads
E14.5_ Qk fl/fl_#1	83,931,860	28,953,888	68.9	13,050,933
E14.5_Qk fl/fl_#2	80,674,030	26,878,815	66.6	11,895,843
E14.5_Nes Qk fl/fl_#1	79,970,980	29,534,795	73.8	13,383,801
E14.5_Nes Qk fl/fl_#2	80,218,216	28,907,399	72.1	12,642,383

**Table 2:** Genes ontology terms associated with Qki-regulated alternative splicing targets.

Term	Description	Log P	Log (*q*-Value)	InTerm_InList	Symbols
GO:0061564	axon development	−9.397	−5.617	26/468	Aatk,Apc,Ddr1,Dab1,Dst,Dvl1,Evl,Fn1,Nfib,Nin,Numb,Ptprf, Ptprm,Ptprs,Ptprz1,Cyfip1,Tnc,Tsc2,Golga4,Brsk2,Foxp1,Boc, Picalm,Nrcam,Auts2,Kalrn,Cask,Cln3,Dab2,Lrp8,Nrxn1,Dennd5a, Map4k4,Nckap1,Grip1,Ccp110,Ccdc88a,Flna,Bcas3,Ripor2,Ttc3, Prpf40a,Actn4,Qrich1,Fmnl2,Triobp,Zmym5,Fat1,Mff,Bnip2, Tenm4,Clstn1,Adgrl2,Adgrb1,Sorbs2,Celf1,Pcnt,Pkm,Zfx,Brd4, Kmt2c,Ankrd26,Sbf1,Atad3a,Hp1bp3,Slc12a7,Arfip2,Cacna1g,Pla2g6
GO:1990778	protein localization to cell periphery	−8.110	−4.934	19/291	Cask,Cln3,Dab2,Flot2,Kif13a,Myo5a,Nrxn1,Numb,Sorbs1,Snap23, Tsc2,Golga4,Clip1,Rer1,Slmap,Flna,Picalm,Dennd4c,Kalrn,Dvl1, Reep2,Apc,Gcc2,Ccdc88a,Bcas3,Ripor2,Sec16a
GO:0007017	microtubule-based process	−7.738	−4.630	31/770	Apc,Bnip2,Cln3,Dctn1,Dst,Dvl1,Dyrk1a,Kif13a,Kif16b,Kif21a,Mark3, Mdm1,Myo1b,Myo5a,Nin,Pcnt,Fbxw5,Clip1,Haus2,Camsap3,Gcc2, Eml2,Brsk2,Eml4,Ccp110,Phldb1,Ccdc88a,Flna,Bcas3,Ripor2,Prc1, Evl,Cyfip1,Nckap1,Afap1,Arfip2,Triobp,Mtss1,Fat1,Sorbs1,Actn4, Foxp1,Sorbs2,Nrxn1,Clstn1,Adgrl2,Adgrb1,Auts2,Pla2g6,Hnrnpa2b1, Mff,Pot1a,Ddhd1,Eif4g3
GO:1903044	protein localization to membrane raft	−6.406	−3.430	4/6	Flot2,Tsc2,Clip1,Reep2,Snap23,Cln3,Dctn1,Dvl1,Nrxn1,Rer1, Tmem175,Adgrb1,Ccdc88a,Mtss1,Sec24c,Sec16a,Picalm,Nrcam
GO:0031345	negative regulation of cell projection organization	−5.720	−2.864	13/199	Aatk,Dab1,Dab2,Evl,Nrxn1,Ptprf,Ptprs,Ptprz1,Dennd5a,Tsc2, Map4k4,Ccp110,Flna,Apc,Dyrk1a,Mdm1,Ttc3,Map3k7,Actn4, Camsap3,Eml2,Eml4,Pot1a,Bcas3,Picalm,Foxp1,Npr2

## Supplementary Materials

Supplementary Materials can be found at https://www.mdpi.com/1422-0067/20/5/1010/s1.
