# Supplementary material for: An RNA Switch of a Large Exon of Ninein Is Regulated by the Neural Stem Cell Specific-RNA Binding Protein, Qki5
Source: Int J Mol Sci. 2019 Feb 26;20(5):1010. doi: 10.3390/ijms20051010 (PMC6429586; doi:10.3390/ijms20051010)
Supplement: Supplementary file 1 [file ijms-20-01010-s001.zip › Yano_ijms-433809_Sup_FigS1_TableS1.pdf]

Table S1. Transcript level changes in *Qk* cKO

gene.ids	gene.names	logFC	logCPM	PValue	p.adj
16669	Krt19	-7.81	-1.33	8.01E-10	3.69E-06
20296	Ccl2	-6.31	-2.55	8.45E-05	0.055630428
435766	Tnni3k	-6.23	-2.60	0.000108017	0.063807378
75388	Boll	-6.07	-2.71	0.000146101	0.079330345
20388	Sftpb	-4.30	-2.23	0.000204561	0.085685053
69189	1810033B17Rik	-4.14	-2.36	0.000137195	0.077090197
23925	Kel	-2.37	-0.93	9.61E-05	0.059839741
21819	Tg	-2.30	-0.98	2.11E-05	0.016722719
12821	Col17a1	-2.10	-0.74	5.37E-05	0.038632205
67425	Eps8l1	-1.79	2.18	6.68E-07	0.001256106
20518	Slc22a2	-1.71	-0.30	0.000200396	0.085647291
19317	Qk	-0.64	8.08	9.42E-12	2.17E-07
15452	Hprt	-0.63	5.49	3.81E-07	0.000797325
228482	Arhgap11a	-0.58	6.79	3.74E-08	0.000107813
12444	Ccnd2	-0.36	9.35	0.000192326	0.085647291
21923	Tnc	0.38	6.80	0.000193303	0.085647291
14199	Fhl1	0.40	6.10	2.28E-05	0.017473508
57776	Ttyh1	0.44	7.33	2.97E-06	0.004269489
69219	Ddah1	0.44	6.73	5.46E-06	0.006372177
26451	Rpl27a	0.45	5.41	7.24E-05	0.050552239
18797	Plcb3	0.45	5.25	0.0001635	0.083704564
12140	Fabp7	0.46	9.28	2.35E-06	0.003611677
68977	Haghl	0.47	5.03	0.000181166	0.085647291
105501	Abhd4	0.47	4.93	0.000148069	0.079330345
20090	Rps29	0.47	5.13	0.000197392	0.085647291
14109	Fau	0.48	4.71	0.000237212	0.095447392
320165	Tacc1	0.49	5.49	8.24E-06	0.00862461
18611	Pea15a	0.51	8.53	8.10E-08	0.000186623
320910	Itgb8	0.52	4.37	0.000240296	0.095447392
19943	Rpl28	0.53	5.02	1.37E-05	0.012110225
17751	Mt3	0.55	4.93	1.04E-05	0.009986615
225743	Rnf165	0.55	6.62	1.91E-08	6.27E-05
68836	Mrpl52	0.55	5.58	5.81E-06	0.006372177
319555	Nwd1	0.63	3.55	0.000216601	0.089108037
107747	Aldh1l1	0.63	4.92	7.09E-07	0.001256106
12824	Col2a1	0.63	6.92	2.55E-10	1.47E-06
22329	Vcam1	0.64	5.14	1.18E-06	0.001938561
13040	Ctss	0.64	3.24	0.00010738	0.063807378
71584	Gdpd2	0.70	3.81	9.38E-06	0.009391855
170790	Mlc1	0.70	4.73	7.65E-08	0.000186623
20319	Sfrp2	0.70	3.82	4.60E-06	0.005887622
13618	Ednrb	0.77	5.30	1.36E-10	1.05E-06
30052	Pcsk1n	0.78	3.08	3.68E-05	0.027322341
208501	1810043H04Rik	0.85	1.92	0.00019779	0.085647291
16869	Lhx1	0.96	2.49	0.000133226	0.076731512
320452	P4ha3	0.98	2.46	1.55E-05	0.012720707
214106	4933430I17Rik	1.11	1.31	9.48E-05	0.059839741
100503019	Gm16551	1.35	0.84	1.29E-05	0.011842205
16373	Irx3	1.47	-0.17	0.000192469	0.085647291
18976	Pomc	2.36	0.46	5.71E-06	0.006372177
18088	Nkx2-2	2.83	-0.40	1.45E-05	0.012409002
73720	Cst6	2.99	-1.89	0.000197556	0.085647291
22412	Wnt9b	3.36	-1.96	0.000200753	0.085647291
20669	Sox14	4.05	-0.82	0.000160651	0.083704564
20476	Six6	4.51	0.22	7.70E-09	2.96E-05
26423	Nr5a1	5.08	-0.16	2.97E-11	3.42E-07
268958	Capn11	6.03	1.69	3.32E-06	0.004503776
14347	Fut7	6.24	-2.65	8.27E-05	0.055630428

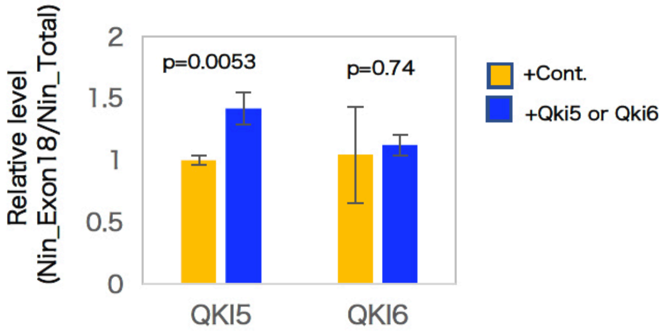
Relative level
(Nin_Exon18/Nin_Total)
2
1.5
1
0.5
0
p=0.0053
p=0.74
QKI5
QKI6
+Cont.
+Qki5 or Qki6

Figure S1
